# Leadership and Motivational Climate: The Relationship with Objectives, Commitment, and Satisfaction in Base Soccer Players

**DOI:** 10.3390/bs9030029

**Published:** 2019-03-19

**Authors:** Concepción Calvo, Gabriela Topa

**Affiliations:** Department of Social and Organizational Psychology, National Distance Education University (UNED), Ph.D. Program in Health Psychology, Madrid 28040, Spain

**Keywords:** leadership, motivational climate, sport, objectives, commitment

## Abstract

The objective of the present study is to analyze non-professional soccer players’ preferences regarding coach leadership style and motivational climate and to determine the relationship of these variables with players’ satisfaction, sport commitment, and sport objectives. The participants were 151 players, aged between 10 and 24 years, divided into five categories: Alevín, Infantil, Cadet, Feminine, and Juvenile, all belonging to the Aragonese Soccer Federation. The participants completed questionnaires assessing their perception of their coach’s leadership style, the team’s motivational climate, their individual satisfaction, degree of sport commitment, and sport objectives. The results show that the leadership styles of training and instruction (M = 3.98, SD = 0.43) and positive feedback (M = 4.02, SD = 0.53) are the most valued by the players in all categories. The training and instruction leadership style had the highest correlations with task-oriented motivational climate (r = 0.40). The findings of the regression analysis show that a training and instruction leadership style and a task-oriented motivational climate significantly predict players’ satisfaction (13.3%) and sport commitment (14.5%).

## 1. Introduction

A large number of children and adolescents dedicate a significant part of their time to the practice of sports activities that are carried out in teams under the direction of a coach. Young people’s experiences, both positive and negative, with a coach’s leadership style and the team’s motivational climate can have a determining influence on their lives [1]. Fortunately, in recent years there has been increasingly more scientific research producing new contributions on leadership styles and motivational climate in team sports [2,3,4,5].

Following the approach of the multidimensional model of Chelladurai, the most widely used model applied to sports as it was fully developed within the sporting context, we consider that leadership is very relevant in team sports because the team members must work together to achieve their goals. The coach provides athletes a direction in their sport activity, while trying to create a positive climate of getting on well. For this purpose, coaches must manage the interpersonal relationships between team members and influence the motivational aspects that affect each athlete. Coaches only become leaders when their athletes accept their authority and ability to wield power over them and when they are respected by the athletes. Successful coaches can motivate their athletes and make them feel that they contribute effectively to the team’s progress [6]. Therefore, coach leadership style is essential to team functioning and can determine whether or not the teams achieve their goals [7].

On the other hand, followers often differ in their desire or their need for leadership. Thus, some athletes accept responsibilities and some autonomy in decision-making, whereas others feel better when they are directed. Depending on age, some studies indicate that young people prefer democratic and social support behaviors in their leaders and reject autocratic behaviors [8]. However, empirical works have not sufficiently clarified whether players prefer democratic, social support, positive feedback, and training and instruction leadership styles, or whether they reject autocratic behaviors. Nor is there enough evidence about the relationship of these preferences and the team’s motivational climate. Finally, it seems relevant to determine if leadership based on training and instruction and a task-oriented motivational climate favors desirable outcomes such as team union [9,10], player satisfaction, and persistence in the activity. The findings of this study will help to understand if leadership style and motivational climate enhance players’ effort and contribute to generating satisfaction and commitment among team members, favoring the achievement of their goals and their persistence in the sport.

### 1.1. Sport Leadership

The multidimensional leadership model of Chelladurai [11] is one of the most frequently applied to the field of sports, and it is the theoretical framework of many empirical studies [12]. According to this model, there are different leadership styles that coaches can put into practice and that coaches should select depending on the characteristics of their subordinates. These leadership styles are: training and instruction, in which coaches try to improve skills, techniques, and strategies and facilitate rigorous training; democratic behavior, in which athletes are allowed to participate in decisions about the group’s goals; autocratic behavior, in which coaches’ personal authority and independent decision-making are emphasized; social support, where coaches consider the individual welfare of the athletes and establish a pleasant relationship with them; and positive feedback, in which athletes are praised or rewarded for their good performance through contingent positive comments on sport performance. 

Previous studies have shown that leadership styles oriented to training and instruction, positive feedback, social support, and democratic behavior are positively related to self-efficacy, sense of competence, satisfaction, and self-esteem in sports [13,14]. More recent work indicates that leadership style is related to cooperative behaviors in team sports [9] among adult athletes. However, despite the increase in empirical research in Spain [15,16,17], studies that confirm whether coaches’ leadership styles are related to athletes’ preferences and sport performance among children and adolescents are still lacking. 

### 1.2. Motivational Climate

From the goal perspective theory, it is argued that the situation created by the coaches, called the motivational climate, can influence the probability of the athletes’ being involved with the ego or with the task in their sport activity [18,19]. When the motivational climate focuses on the task, the athletes’ main objective is to perform with mastery, gain skills and knowledge, put out maximum effort, and give the best of themselves [1,3,19]. Conversely, when the climate is centered on the ego, the players are concerned with their own ability and with showing that they are more competent than others [1,5,10,19,20]. Therefore, they believe they are successful when they think they perform better or the same as others, but with less effort. The task-oriented motivational climate—in which coaches’ value and encourage effort development and cooperation among team members and consider that mistakes are part of learning—promotes players’ psychological well-being. Studies show that players express less anxiety, greater confidence, and higher self-esteem [20,21]. Likewise, the task-oriented climate favors performance and the reduction of antisocial behaviors [22,23].

When coaches favor the ego-oriented climate—in which mistakes are punished, interpersonal competition and rivalry between the team members is enhanced, and only players with high capacity stand out—there is less psychological well-being, more anxiety about performance, and less satisfaction in the sport environment [24].

In the present study, players’ satisfaction, players’ degree of sport commitment, and players’ sport objectives are considered the outcome variables, since they have been included in most of the previous empirical studies as indicators of personal well-being and performance.

On the basis of the literature reviewed, the multidimensional model of leadership in sports [11,16,25], and the socio-cognitive theory of meta-perspectives [23,24], the hypotheses of this study are:

**Hypothesis** **1 (H1):** Players will prefer democratic leadership and social support styles and will reject autocratic behaviors.

**Hypothesis** **2 (H2):** Players’ preferences for perceived leadership style and motivational climate will positively predict their objectives, degree of commitment, and satisfaction in the sport setting.

## 2. Method

### 2.1. Participants

We used a sample of 151 soccer players, divided into five categories—Juvenile: 40 players (26.50%); Female: 17 players (11.30%); Cadet: 45 players (29.80%); Infantil: 32 players (21.20%); and Alevín: 17 players (11.30%). All the players were male, except the players for the Female and Infantil teams were mixed. All the teams were trained by male personnel. The players’ ages ranged between 10 and 24 years (M = 15.59, SD = 2.38).

Concerning educational level, 56.95% of the players were studying compulsory secondary education (CSE), 21.85% were enrolled in high school, 11.92% were enrolled in primary education, 4.64% were enrolled in university studies, 2.65% were enrolled in middle-degree vocational training, 0.66% were enrolled in high-degree vocational training, and 1.32% were not currently studying.

Concerning their experience in the clubs, 41.72% had been playing in the club between 3 and 5 years, 30.46% for more than 5 years, 13.91% between 1 and 2 years, and for 13.91% this was their first year at the club. The percentage of training absenteeism was low; 66.67% missed 0–5 workouts throughout the season, 22.67% missed 6–10 training sessions, 6% missed 11–15 training sessions, and 4.67% missed more than 15 training sessions, with the most commonly mentioned reasons being injuries or exams.

### 2.2. Instruments

Players’ preferences for leadership style: The Leadership Sport Scale (LSS) [11] was used to assess this variable, in the Spanish version adapted to soccer [25] made up of 40 items that were distributed in five subscales. In order to evaluate the players’ preferences, the questionnaire began with the stem phrase “I prefer my coach to...”, and then the coach’s behaviors were described. Two of the subscales measured the coaches’ styles of decision-making and democratic behavior (nine items) and autocratic behavior (five items). Another two subscales measured the coaches’ motivational tendencies social support (eight items) and positive feedback (five items). The fifth subscale measured the coaches’ training and instruction behavior (13 items). Previous studies have provided evidence for the reliability of the scale [5,9]. In the present study, the reliability of the instrument was adequate, ranging between 0.74 for the training and instruction dimension and 0.63 for democratic behavior.

Motivational climate: The Perceived Motivational Climate in Sport Questionnaire-2 was used to assess this variable, in the Spanish version adapted to soccer [26], made up of 29 items that were distributed in two subscales, task-oriented climate and ego-oriented climate. The task-oriented climate contained three factors: cooperative learning, effort/improvement, and the importance of the role of the player within the team. The ego-oriented climate was made up of three factors which included punishment for mistakes, unequal recognition, and rivalry among team members. In previous studies, the scale showed its reliability [1] and in the present study the Cronbach alpha values were 0.88 for the task-oriented climate and 0.71 for the ego-oriented climate.

Players’ satisfaction: The Intrinsic Sport Satisfaction Questionnaire [27] was used to assess this variable, in the Spanish version [28] made up of seven items, divided into two subscales, satisfaction/fun and boredom. In previous studies with a Spanish population, the scale showed adequate psychometric properties [28], and in the present study its reliability was 0.78.

Players’ degree of sport commitment: The Degree of Sport Commitment Scale [29] was used to assess this variable, made up of 11 items that measured current commitment and future commitment. Previous validation studies in Spanish showed the psychometric properties of the scale [30], which in the present study had a reliability of 0.77.

Players’ sport objectives: The Goal Content for Exercise Questionnaire (GCEQ) [31] was used to assess this variable, made up of 19 items that measured social affiliation, image, health management, social recognition, and skill development. In previous studies, this instrument showed adequate reliability [32,33]. In the present study, the reliability ranged between 0.87 for social recognition and 0.69 for skill development.

All instruments were rated on a five-point Likert scale ranging from 1 (strongly disagree) to 5 (strongly agree).

Sociodemographic information was collected through questions inserted at the beginning of the questionnaire, relating to age, gender, level of studies in progress, category in which the sport was practiced, time, and number of hours dedicated to sport practice.

### 2.3. Procedure

The present study was approved by the Ethics Committee of the UNED through a protocol issued on 5 April 2017. The participants of the study were part of the two sports organizations of base soccer for the population of Calatayud (Zaragoza), and all the players were registered in the Aragonese Soccer Federation and duly federated in their respective categories. Prior consent was requested from the directors of the participating clubs. Before completing the questionnaire, prospective participants were informed of the purpose and objectives of the study both verbally and in writing, and they gave their signed consent on the back of the study fact sheet. In addition, as the participants were mostly minors, their parents were informed in writing, and were requested to sign the appropriate consent for their children to participate in the study.

The questionnaires were completed by the players in April, which was almost at the end of the season, as they had started competing in August of the previous year. This time margin was considered appropriate for the players and coaches to have shared time and space and to have had the opportunity to adapt to and get to know each other. About 10% of the players refused to participate in this voluntary study. We also excluded those who expressed their intention to take part in the study but did not provide their parent’s duly completed and signed informed consent. The questionnaires were filled out by the participants in the club facilities, before one of the routine trainings. The players were given the necessary material to complete the surveys. A member of the research group, an experienced psychologist, stayed with them to clarify their doubts, assuring the participants of the anonymity and confidentiality of the results. The questionnaires were collected in the same dressing room where they were completed.

## 3. Data Analysis

A descriptive analysis of the scales was performed. The Cronbach alpha of each scale was obtained: Leadership (α = 0.81), task climate (α = 0.89), ego climate (α = 0.73), satisfaction (α = 0.78), degree of commitment (α = 0.78), and sport objectives (α = 0.91). The Pearson correlations between the dimensions of the different study variables were calculated. Finally, a linear regression analysis was performed to confirm whether the relationships found between the study variables significantly predict the outcome variables. The statistical program SPSS 25.0 was used to analyze the data.

## 4. Results

Table 1 shows the means of the dimensions of the LSS. The players preferred the coaches’ leadership styles of positive feedback and training and instruction behavior, followed by social support and democratic behavior, with autocratic behavior being the least preferred by the athletes.

As can be seen in Table 1, all the styles significantly correlated with each other, except for autocratic behavior. The highest correlations were observed between training and instruction behavior and positive feedback (*r* = 0.54), training and instruction behavior and democratic behavior (*r* = 0.46), social support and positive feedback (*r* = 0.39), and training and instruction behavior and social support (*r* = 0.38). Significant correlations were also found between democratic behavior and social support (r = 0.33), and between democratic behavior and positive feedback (*r* = 0.34). On the other hand, democratic behavior and autocratic behavior correlated with each other (r = 0.10), although this correlation did not reach statistical significance. A task-oriented motivational climate was positively related to the leadership styles training and instruction behavior (r = 0.40), democratic behavior (r = 0.25), social support (r = 0.38), and positive feedback (r = 0.26), whereas autocratic behavior had a negative and significant relationship with this motivational climate (r = 0.40).

The results show that the leadership styles perceived by the players were positively and significantly related to their satisfaction in the sporting environment, training and instruction behavior (r = 0.31), social support (r = 0.22), and positive feedback (r = 0.23). The relationship between democratic behavior and satisfaction did not reach statistical significance, whereas autocratic behavior showed a negative correlation with satisfaction (r = −0.18). The only leadership dimension significantly associated with sport commitment was training and instruction behavior (*r* = 0.26). Sport objectives only had a positive and significant relationship with democratic behavior (*r* = 0.21). Lastly, task-oriented motivational climate was positively and significantly related to the three outcome variables: satisfaction (*r* = 0.30), sport commitment (*r* = 0.31), and sport objectives (*r* = 0.28).

The results of stepwise multiple linear regression analyses (see Table 2) supported the proposed hypotheses. In relation to the degree of satisfaction, in the first step of the regression equation, leadership style based on training and instruction was significant and in the second step, task-oriented motivational climate was significant. In relation to the coefficient of explained variance (*R^2^*), in Model 1, training and instruction leadership style predicted 9% of total satisfaction, and in Model 2, task-oriented climate increased the explained variance to 13.3%. In relation to players’ sport commitment, in the first step of the equation, coaches’ training and instruction leadership style was significant, explaining 8% of the variance. In Model 2, task-oriented motivational climate was added and was significant, explaining 14.5% of the variance. In relation to sport objectives, in the first regression step, the only significant predictor was task-oriented motivational climate, explaining 8% of the variance.

## 5. Conclusions 

To summarize, the preceding studies provide support for the relationship found in our study on players’ preference for the leadership styles of training and instruction and positive feedback. The results showed that the players preferred training and instruction leadership styles [34,35,36,37,38,39,40] and positive feedback [38,41,42,43,44], which indicates that the players show a learning attitude towards sport rather than a recreational attitude, contrary to what one might think.

As for autocratic behavior, it is the least desired [37,40,41,42,44] and implies less satisfaction of the players [38,45], which shows that for our teams, a participatory, cooperative, and communicative environment is promoted among all members.

Our data do not show the tendency of other studies that report that the training and instruction [38,45] and democratic styles [38,42] correlate positively with players’ satisfaction. In fact, democratic behavior is the only leadership style in our study that did not show a significant correlation with players’ satisfaction.

Regarding the motivational climate preferred by players, it is based on the task engagement, coinciding with the results of Martens [46], and the most important situational variable is the task to be done. This gives the players greater feelings of overall satisfaction as opposed to ego orientation [47,48,49,50,51,52,53], as well as being considered as a good predictor of sport performance [48,54]. This orientation is very important, especially in team sports, as in our case, because they need more direction, coordination, and structuring of the group. This emphasizes that players’ perception of a task-oriented climate is sought in teams, even in those that require maximum performance, such as official competitions.

In the correlations found, all the leadership styles, except for autocratic behavior, correlate positively and significantly with each other and with a task-oriented motivational climate, supporting our hypothesis. The regression analyses show that the leadership dimension based on training and instruction acts as a predictor of the outcome variables players’ sport satisfaction and sport commitment. The task-oriented motivational climate also predicts players’ satisfaction, sport commitment, and sport objectives. The joint analysis of these variables (task-oriented motivational climate and training and instruction style) better predicts the dependent variables of players’ satisfaction and sport commitment, with a greater weight for the task-oriented motivational climate.

## 6. Discussion

Although we have tried to deepen the analysis of the relationships with the different target variables, this research presents some limitations. First, it is a cross-sectional study and, although several authors in previous studies have reported results of correlations and reliability similar to those found in this document, longitudinal studies should be carried out and the relationships with the different leadership styles and motivational climate should be examined in depth. This would provide information about the stability of the results obtained.

In the present study, data were collected through self-report questionnaires. Although we took the precaution to not place players and coaches in the same space when completing their questionnaires, the answers may be biased in some cases due to social desirability, especially in younger age groups.

To analyze the influence of the motivational climate on players at these ages other factors that may affect them should be considered, such as parents, classmates, family situation, or social status. This would be an interesting pathway for future research.

In short, the objective of effective leadership is to create and develop a climate within the organization or team that will achieve the desired performance and the satisfaction expected by each of the team members. On the basis of the results obtained, we encourage parents, coaches, and sports technicians of young athletes to instill values based on effort, work, cooperation, and support, thus creating in the group a climate that favors communication and a good feeling among team members. In addition, they should reject individualistic behaviors that seek personal recognition and satisfaction at the expense of teamwork. Those responsible for the instruction of young athletes should be flexible in adopting the appropriate leadership style for each situation and the team’s own culture, which unites all the members around the same goals.

## Figures and Tables

**Table 1:** Pearson’s correlation matrix of study variables.

Variables	Mean	S.D.	1	2	3	4	5	6	7	8	9	10
1. Training and instruction	3.98	0.43	1									
2. Democratic behavior	3.45	0.51	0.46 **	1								
3. Autocratic behavior	2.57	0.86	−0.07	0.10	1							
4. Social support	3.70	0.58	0.38 **	0.33 **	0.09	1						
5. Positive feedback	4.02	0.53	0.54 **	0.34 **	−0.07	0.39 **	1					
6. Task-oriented climate	4.10	0.50	0.40 **	0.25 **	−0.16 *	0.38 **	0.26 **	1				
7. Ego-oriented climate	2.80	0.82	−0.07	0.03	0.23 **	−0.24 **	−0.02	−0.44 **	1			
8. Satisfaction with sport practice	4.43	0.50	0.31 **	0.16	−0.18 *	0.22 **	0.23 **	0.30 **	−0.05	1		
9. Sport commitment	4.03	0.54	0.26 **	0.08	−0.03	0.14	0.09	0.31 **	−0.02	0.50 **	1	
10. Sport goals	3.55	0.67	0.13	0.21 **	−0.05	0.13	0.07	0.28 **	−0.05	0.07	0.25 **	1

Note: ** *p* < 0.01; * *p* < 0.05.

**Table 2:** Regression analyses for satisfaction with sport, sport commitment, and sport goals.

	Criterion Variables				
	Satisfaction with Sport		Sport Commitment		Sport Goals
Predictor Variables	Model 1	Model 2	Model 1	Model 2	Model 1
First step: Coaches’ leadership					
Training and instruction	0.306 **	0.219 **	0.302 **	0.222 *	n.s.
Second step: Motivational climate					
Task-oriented climate		0.217 **		0.326 **	0.283 **
F (df)	15.35 (1.149)	11.33 (2.148)	2.43 (1.149)	3.46 (2.14)	12.95 (1.149)
R^2^	0.093	0.133	0.078	0.145	0.080

Note: df: degrees of freedom; n.s.: non-significant, ** *p* < 0.01; * *p* < 0.05.
